# Study and analysis of the correlation between lumbar spondylolisthesis and Modic changes

**DOI:** 10.3389/fsurg.2024.1296275

**Published:** 2024-02-07

**Authors:** Guang-qing Li, Xiao Kang, Wei Li, Shi-shen Pei

**Affiliations:** ^1^ Spine Surgery Department, The Fourth People’s Hospital of Hengshui, Hengshui, China; ^2^ Imaging Department, The Fourth People’s Hospital of Hengshui, Hengshui, China

**Keywords:** lumbar spondylolisthesis, Modic changes, intervertebral disc degeneration, stress-induced angle, stress concentration effect

## Abstract

**Background:**

This study aimed to explore the risk factors of Modic changes in lumbar spondylolisthesis.

**Methods:**

The distribution of Modic changes in different types of lumbar spondylolisthesis, degree of spondylolisthesis, and degree of intervertebral disc degeneration in patients with lumbar spondylolisthesis was observed and analyzed. Statistical analysis was conducted to assess whether intervertebral disc degeneration, local mechanical changes, etc. affect the occurrence of Modic changes. The risk factors of Modic changes in lumbar spondylolisthesis were further illustrated.

**Results:**

The age in the lumbar spondylolisthesis with Modic changes group was younger than that in the lumbar spondylolisthesis without Modic changes group, and the bone mineral density was better in the lumbar spondylolisthesis with Modic changes group than that in the lumbar spondylolisthesis without Modic changes group, *P* < 0.05. The two groups statistically differed in intervertebral disc height (IDH) and disc angle on magnetic resonance imaging (MRI). In the classification of Modic changes, the incidence of type II was the highest. The incidence of Modic changes is higher in isthmic spondylolisthesis than in degenerative spondylolisthesis. With the aggravation of lumbar spondylolisthesis and intervertebral disc degeneration, the incidence of Modic changes gradually increased. Modic changes are most commonly seen in both the upper and lower endplates. Logistic regression analysis showed that the occurrence of Modic changes in lumbar spondylolisthesis was significantly correlated with IDH, disc angle on MRI, type of spondylolisthesis, degree of spondylolisthesis, and degree of intervertebral disc degeneration, *P* < 0.05.

**Conclusions:**

The occurrence of Modic changes is related to the type of spondylolisthesis, the degree of spondylolisthesis, the degree of disc degeneration, the decrease of intervertebral disc height, and local stress angulation.

## Introduction

Lumbar spondylolisthesis is a common clinical disease. It is characterized by partial slippage of adjacent vertebral bodies, leading to uneven lumbar body sequence. Isthmic spondylolisthesis and degenerative spondylolisthesis are more common. In 1955, Newman concluded that most cases of lumbar spondylolisthesis were accompanied by degenerative changes in the intervertebral discs and small vertebral joints, and they were the first to propose the concept of degenerative lumbar spondylolisthesis (1). Degenerative lumbar spondylolisthesis is the most common clinical lumbar spondylolisthesis; it is also known as pseudospondylolisthesis because the isthmus of the arch remains intact. Isthmic lumbar spondylolisthesis has a fracture in its isthmus and is also known as true spondylolisthesis. Modic changes (MCs) refer to abnormal signal changes in the vertebral endplate and subendplate bone on magnetic resonance imaging (MRI). In 1987, de Roos et al. first reported that abnormal signals in the adjacent vertebral endplate region were found in MRI scans of patients with lumbar degenerative disease (2). In 1988, Modic et al. systematically described the types, classification criteria, and histological changes associated with this signal change (3). Modic believes that the root cause of Modic changes is intervertebral disc degeneration. When the degeneration of the intervertebral disc leads to the decrease of the protective effect of the endplate, the bone under the endplate is gradually destroyed, and edema, fat infiltration, fibrosis, and sclerosis gradually appear, thus showing different signal changes on MRI (3, 4). Hayashi et al. found that age, disc degeneration, lumbar angular motion, and horizontal motion were all related to the occurrence of Modic changes in the lumbar dynamic MRI study of 450 patients with lumbar symptoms (5).

In clinical practice, it is not uncommon to find Modic changes in patients with lumbar spondylolisthesis However, no studies have reported the risk factors for Modic changes in lumbar spondylolisthesis. It is of great significance to clarify the imaging changes, such as intervertebral disc degeneration and Modic changes, in patients with lumbar spondylolisthesis, which is of great significance for delaying the progression of the disease and early prevention and treatment. In this study, we compared the clinical and imaging data of patients with isthmic and degenerative lumbar spondylolisthesis to explore the risk factors of Modic changes.

## Materials and methods

The basic information and imaging examination results of 719 patients with lumbar spondylolisthesis in our hospital from January 2014 to June 2023 were retrospectively analyzed. The inclusion criteria were the following: (1) L4 or L5 anterior spondylolisthesis and single spondylolisthesis; (2) occurrence of Modic changes in the corresponding segment of lumbar spondylolisthesis; (3) isthmic or degenerative lumbar spondylolisthesis; and (4) availability of complete imaging data. The exclusion criteria were the following: (1) non-L4 or L5 slippage; (2) double slippage or multiple slippages; (3) posterior or lateral slippage; (4) L4 or L5 vertebral lesions such as hemangioma, primary malignant tumor, metastatic vertebral tumor, etc.; (5) previous history of lumbar surgery; and (6) presence of Modic changes but in non-lumbar spondylolisthesis segments. Based on the inclusion and exclusion criteria, 626 patients with lumbar spondylolisthesis were finally eligible. There were 150 patients with lumbar spondylolisthesis with Modic changes, forming the MCs group. A total of 150 patients without Modic changes in lumbar spondylolisthesis were randomly selected, forming the non-Modic changes (NMCs) group. This study was approved by the Ethics Committee of Hengshui Fourth People's Hospital, and informed consent was obtained from all participants.

## Evaluating indicators

### Basic patient information

General baseline data such as gender, age, bone mineral density (BMD), body mass index (BMI), and onset time were compared between the MCs group and the NMCs group.

### Radiographic analyses

**Figure 1:**
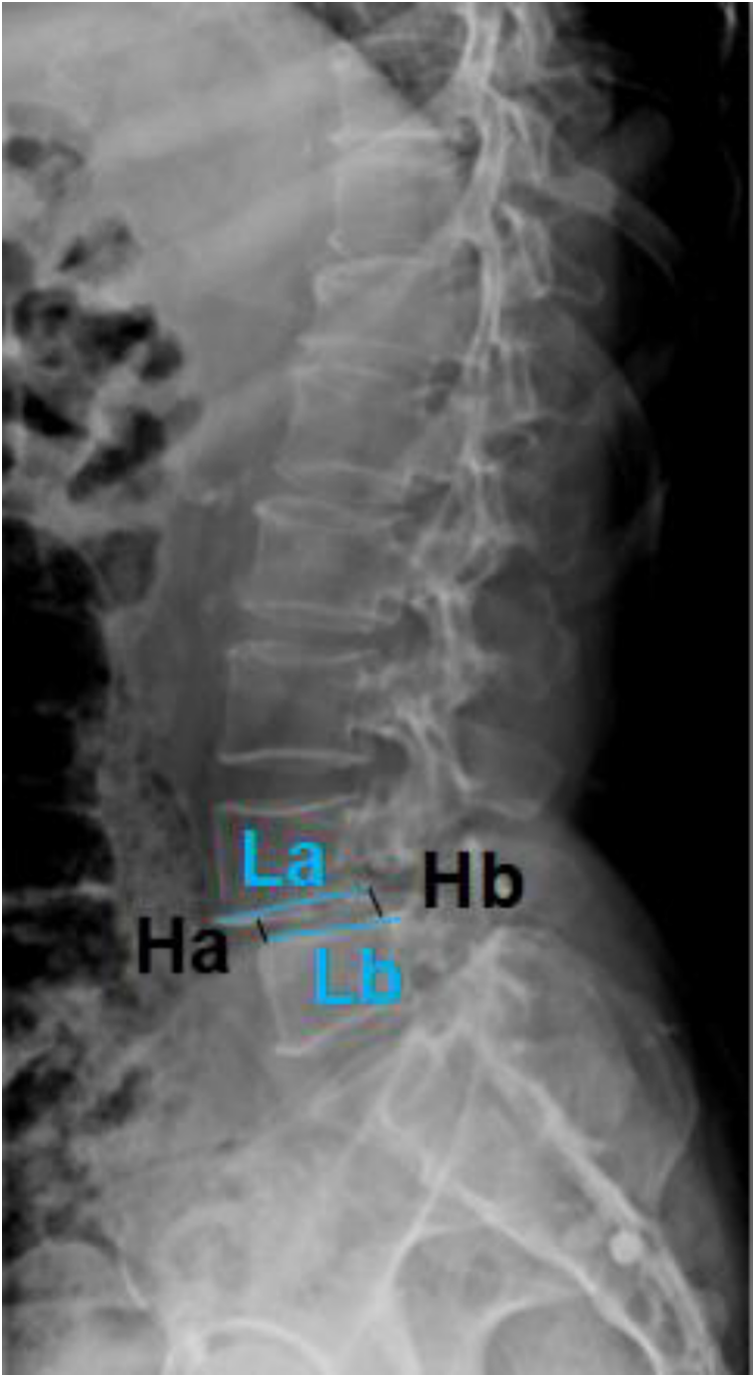
IDH = (Ha + Hb)/(La + Lb).

All radiological parameters were measured by a radiologist and a spine surgeon and averaged. The agreement was classified by *k* values: 0–0.2 for slight, 0.21–0.4 for fair, 0.41–0.60 for moderate, 0.61–0.8 for substantial, and 0.81 or more for excellent. Sagittal parameters such as pelvic incidence (PI), pelvic tilt (PT), sacral slope (SS), lumbar lordosis (LL), and intervertebral disc height (IDH) were determined based on lumbar anteroposterior and lateral x-rays or whole spine anteroposterior and lateral x-rays. At the same time, the location, type, and degree of lumbar spondylolisthesis were determined. Due to the existence of individual differences, the IDH measurement method is established by Lan et al., as shown in Figure 1, that is, IDH = (anterior intervertebral disc height + posterior intervertebral disc height)/(upper endplate length + lower endplate length), reducing the error caused by individual differences (6). The degree of lumbar spondylolisthesis was determined according to the Meyerding index (7). The upper endplate of the lower vertebral body was divided into four equal parts on average. According to the degree of spondylolisthesis of the upper vertebral body to the lower vertebral body, it was divided into four degrees: degree 1, the vertebral body forward slipped not more than a quarter of the lower vertebral body; degree 2, the vertebral body forward slipped no more than the midsagittal diameter of the vertebral body, that is, one-half; degree 3, the vertebral body forward slipped more than half but not more than three-quarters; and degree 4, the vertebral body forward slipped more than three-quarters of the vertebral body.

**Figure 2:**
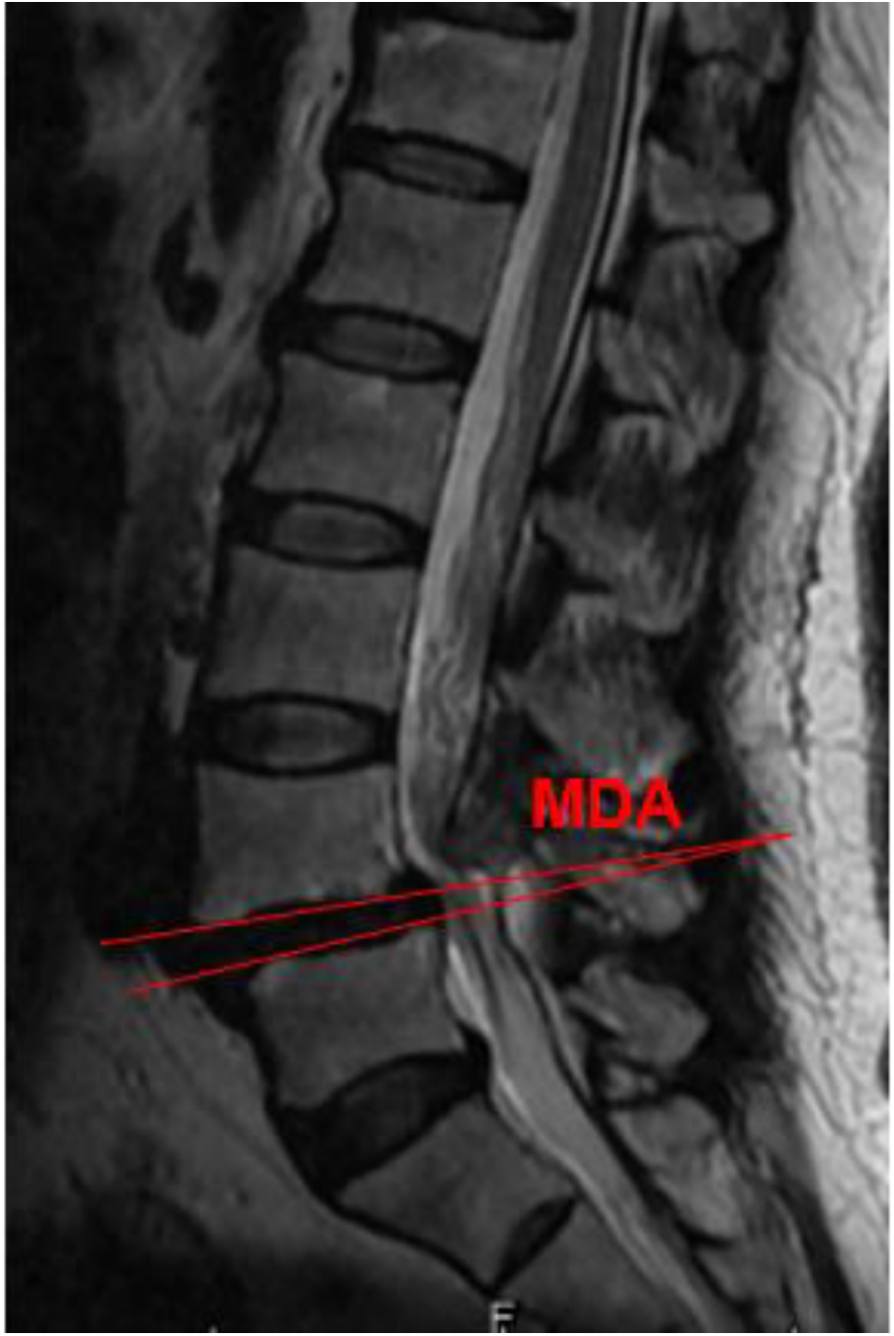
L4 was grade I spondylolisthesis, and MDA was the angle between L4 and L5 on MRI.

According to MRI, the disc angle at the slip site was determined, that is MDA, as shown in Figure 2. At the same time, according to the classification of Modic changes, the type and location of Modic changes were determined. Modic changes classification (3, 8, 9): Type I : T1 showed low signal and T2 showed high signal, which were acute inflammatory changes in subchondral bone marrow, such as edema, congestion and leukocyte infiltration. Type II : T1 and T2 both showed high signal, which was lipid replacement in subchondral bone marrow. Type III : T1 and T2 both showed low signal, extensive bone sclerosis, almost no residual bone marrow. According to the modified Pfirrmann classification of intervertebral disc degeneration proposed by Griffit et al. (10), the degree of intervertebral disc degeneration at the slip site was determined on MRI. Finally, Logistic regression analysis was used to analyze the risk factors of Modic changes.

### Statistical analysis

All statistical analyses were performed using SPSS software (Version 26.0, Chicago, IL, USA). The concordance between the two raters was examined using the Kappa concordance test. The Kolmogorov–Smirnov test was used to examine the normal distribution within the groups. For continuous variables that conform to the normal distribution, a two-sample *t*-test was used for comparison between groups. For continuous variables that do not conform to the normal distribution, the Mann–Whitney *U* test was used for intergroup comparison. The chi-squared test or Fisher's exact test was used to compare categorical variables between groups. Logistic regression analysis was used to evaluate the risk factors of Modic changes. *P* < 0.05 was considered statistically significant.

## Results

There was no significant difference in gender, BMI, and onset time between the MCs group and the NMCs group. However, the patients in the MCs group were younger than those in the NMCs group, and the BMD of the MCs group was better than that of the NMCs group. The difference was statistically significant, as shown in Table 1. Furthermore, interobserver agreement was excellent for both readers, with kappa values ranging from 0.81 to 0.90.

**Table 1:** Comparison of baseline patient data.

	MCs group (*n* = 150)	NMCs group (*n* = 150)	*P*-value
Gender (M/F)	57/93	44/106	0.112
Age (years)	53.44 ± 9.80	59.68 ± 9.66	<0.001*
BMD	−0.90 ± 1.04	−1.70 ± 1.09	<0.001*
BMI (kg/m^2^)	25.62 ± 4.00	26.02 ± 3.83	0.252
Onset time (months)	47.92 ± 60.31	53.74 ± 63.94	0.438

M, male; F, female.

* *P* < 0.05.

There was no significant difference in PI, PT, SS, and LL between the MCs group and the NMCs group. However, IDH in the MCs group was smaller than that in the NMCs group, and MDA was larger than that in the NMCs group, *P* < 0.05, see Table 2.

**Table 2:** Comparison of sagittal parameters.

	MCs group (*n* = 150)	NMCs group (*n* = 150)	*P*-value
PI	56.23 ± 12.69	58.94 ± 10.92	0.161
PT	22.11 ± 11.20	23.31 ± 10.64	0.500
SS	36.81 ± 9.90	35.51 ± 8.76	0.322
LL	44.52 ± 10.63	46.15 ± 11.45	0.425
IDH	16.85 ± 5.42	24.01 ± 6.63	<0.001*
MDA	8.92 ± 4.86	6.25 ± 3.54	<0.001*

* *P* < 0.05.

There were 70 cases and 80 cases of lumbar spondylolisthesis at L4 and L5 in the MCs group and 120 cases and 30 cases in the NMCs group, respectively. The difference was statistically significant. There were 47 cases of degenerative spondylolisthesis and 103 cases of isthmic spondylolisthesis in the MCs group and 125 cases of degenerative spondylolisthesis and 25 cases of isthmic spondylolisthesis in the NMCs group. The difference was statistically significant. There were 71, 68, and 11 cases in the MCs group and 120, 28, and 2 cases in the NMCs group of lumbar spondylolisthesis of degree I, II, and III, respectively, with *P* < 0.05, and the difference was statistically significant. In intervertebral disc grading, the difference between the MCs and NMCs groups in II, III, IV, VI, VII, and VIII grades was statistically significant, *P* < 0.05. With the aggravation of intervertebral disc degeneration, the number of patients in the MCs group increased gradually compared with the NMCs group, as shown in Table 3.

**Table 3:** Lumbar spondylolisthesis and lumbar disc degeneration grade statistics.

	MCs group (*n* = 150)	NMCs group (*n* = 150)	*P*-value
Slip segment (L4/L5)	70/80	120/30	<0.001*
Type of slip (D/I)	47/103	125/25	<0.001*
Degree of slip			
I	71	120	<0.001*
II	68	28	<0.001*
III	11	2	0.011*
Intervertebral disc degeneration grading			
II	3	28	<0.001*
III	8	47	<0.001*
IV	17	41	<0.001*
V	26	22	0.529
VI	13	4	0.025*
VII	27	5	<0.001*
VIII	56	3	<0.001*

D/I, degenerative/isthmic.

* *P* < 0.05.

In the classification of Modic changes, there were 18, 38, and 14 cases of type I, type II, and type III in the L4–5 segment , respectively, totaling 70 cases. There were 16 cases of type I, 57 cases of type II, and 7 cases of type III in the L5–S1 segment, totaling 80 cases. There were 34, 95, and 21 cases of type I, type II, and type III Modic changes, respectively. Type II Modic changes were the most common, see Table 4.

**Table 4:** Modic changes statistics.

	Modic changes type			Total
	I	II	III	
L4–5	18	38	14	70
L5–S1	16	57	7	80
Total	34	95	21	150

Among the Modic changes in the endplate, there were 8, 16, and 126 cases of the single upper endplate, single lower endplate, and coexistence of upper and lower endplates, respectively, and coexistence of upper and lower endplates was the most common, as shown in Table 5.

**Table 5:** Modic changes statistics in the endplate.

	Modic changes exist in the endplate			Total
	Single upper endplate	Single lower endplate	Coexistence of upper and lower endplates	
L4–5	5	12	54	71
L5–S1	3	4	72	79
Total	8	16	126	150

Logistic regression analysis showed that the occurrence of Modic changes in lumbar spondylolisthesis was significantly correlated with IDH, MDA, type of spondylolisthesis, degree of spondylolisthesis, and degree of intervertebral disc degeneration, *P* < 0.05, see Table 6.

**Table 6:** Logistic regression analysis.

	Presence of MCs				
Variables	*β*	SE	Wald *X*^2^	OR (95% CI)	*P*-value
Age	−0.014	0.026	0.273	1.014 (0.963–1.066)	0.602
BMD	0.023	0.035	0.405	1.023 (0.954–1.096)	0.524
IDH	−0.090	0.059	2.346	0.914 (0.815–1.025)	0.031*
MDA	0.206	0.057	12.905	1.228 (1.098–1.374)	<0.001*
Slip segment					
L5	0.202	0.642	0.099	1.224 (0.348–4.309)	0.753
L4	—	—	—	—	—
Type of slip					
Isthmus	−1.323	0.716	3.419	0.266 (0.065–1.083)	0.036*
Degenerative	—	—	—	—	—
Degree of slip					
III	2.125	1.658	1.643	8.373 (0.325–215.693)	0.036*
II	1.227	1.585	0.600	3.412 (0.153–76.241)	0.041*
I	—	—	—	—	—
Disc degeneration grading					
VIII	6.899	1.895	13.252	990.985 (24.152–40,660.593)	<0.001*
VII	5.060	1.464	11.939	157.521 (8.932–2,777.936)	0.001*
VI	4.315	1.400	9.495	74.778 (4.807–1,163.144)	0.002*
V	2.360	1.556	2.301	10.588 (0.502–223.375)	0.129
IV	3.466	1.367	6.430	32.001 (2.197–466.186)	0.011*
III	1.282	1.452	0.779	3.603 (0.209–62.058)	0.377
II	—	—	—	—	—

* *P* < 0.05.

## Discussion

Lumbar spondylolisthesis is a common spinal disease in the clinic. It is divided into isthmic spondylolisthesis and degenerative spondylolisthesis according to whether the pedicle isthmus is continuous. Modic changes refer to the signal changes in the spinal endplate and subendplate bone on MRI. It is not uncommon to see Modic changes in patients with lumbar spondylolisthesis. In this study, the incidence of lumbar spondylolisthesis with MCs was 23.96%, which was lower than the incidence of 29.81% reported by Lan et al. (6). The incidence of Modic changes in the normal population is about 12.0%–13.0% (11). The incidence of MCs in the thoracic spine is only 2.5% (12). The incidence of MCs in the cervical spine is similar to that in the lumbar spine (13). Due to the minimum activity of the thoracic spine, the incidence of Modic changes in the spine is the smallest in the thoracic spine, considering that MCs are related to activity.

Wu et al. believed that PI is a risk factor for MCs, and a smaller PI is associated with a higher incidence of MCs (14). This study showed that MCs had no significant correlation with PI, PT, SS, and LL but had a correlation with MDA and IDH, indicating that the occurrence of MCs was not related to the overall change of spinal sagittal position but related to the change of local factors.

Whether or not accompanied by Modic changes, the incidence of lumbar spondylolisthesis in women is higher than in men, which may be related to estrogen. Han et al. also believed that women are more prone to lumbar spondylolisthesis (15). The age of onset of lumbar spondylolisthesis patients with Modic changes is concentrated at 53.44 ± 9.80 years old. This stage is the transition period of women's menopause. The estrogen in the body is disordered and fluctuates greatly, resulting in symptoms or aggravation. In the NMCs group (59.68 ± 9.66 years old), estrogen was at a low level and relatively stable stage, and the effect of estrogen on Modic changes was smaller than that in the MCs group, which could explain why Modic changes were more likely to occur in the menopausal transition. The research made by Liney et al. is consistent with our point of view (16). The bone mineral density of the MCs group was better than that of the NMCs group, which means that the better the bone quality, the higher the probability of MC occurrence, which is consistent with the age of onset shown in this study. However, it is not consistent with the research done by Karchevsky et al. (17).

This study showed that the incidence of Modic changes in isthmic spondylolisthesis was significantly higher than that in degenerative spondylolisthesis, *P* < 0.05. The reason may be related to the loss of stability of the posterior column of the spine caused by the spondylolysis and the reduction of the limitation of the range of motion of the vertebral body by the facet joints on both sides of the upper and lower vertebral bodies (18). In our study, we showed that Modic changes are most common in type II, followed by type I, with type III being the least common, which is consistent with the research conducted by Modic et al. (3) and Guo et al. (19). According to Mitra et al. (20), type I Modic changes may be an acute state of vertebral endplate bone marrow injury caused by intervertebral disc degeneration, while type II may be a chronic or stable state, and type III is caused by intervertebral disc degeneration. Reactive new bone formation after vertebral injury is the final outcome of type I and type II degeneration. According to the results of our research, it is basically the same as the above point of view. Type I Modic changes are in the acute phase, and the main clinical manifestation is low back pain. When the Modic changes further develop to the bone marrow transformation stage, that is, type II Modic changes, the appearance of yellow bone marrow leads to a decrease in the stability of the vertebral body, which makes the lumbar spine more prone to angular displacement and horizontal displacement, resulting in lumbar spondylolisthesis. The occurrence of spondylolisthesis further aggravates the development of Modic changes. This stage lasts for a long time in clinical practice, and the proportion of patients is also the largest. However, with the fibrosis and sclerosis of the yellow bone marrow in the vertebral body, the stability of the lumbar spine significantly enhances after local self-fusion. However, at this stage, MRI often shows significant degeneration of the intervertebral disc, significant reduction of the intervertebral disc height, and often type III Modic changes.

According to our results, the incidence of grade I and II lumbar spondylolisthesis is higher, while the incidence of grade III spondylolisthesis is lower. The incidence of Modic changes in grade I, II, and III lumbar spondylolisthesis was 37.17%, 70.83%, and 84.61%, respectively. With the aggravation of lumbar spondylolisthesis, the incidence of Modic changes gradually increased. Although grade III spondylolisthesis is rare in clinical practice, it is most prone to Modic changes. With the aggravation of intervertebral disc degeneration, the probability of Modic changes also increases.

**Figure 3:**
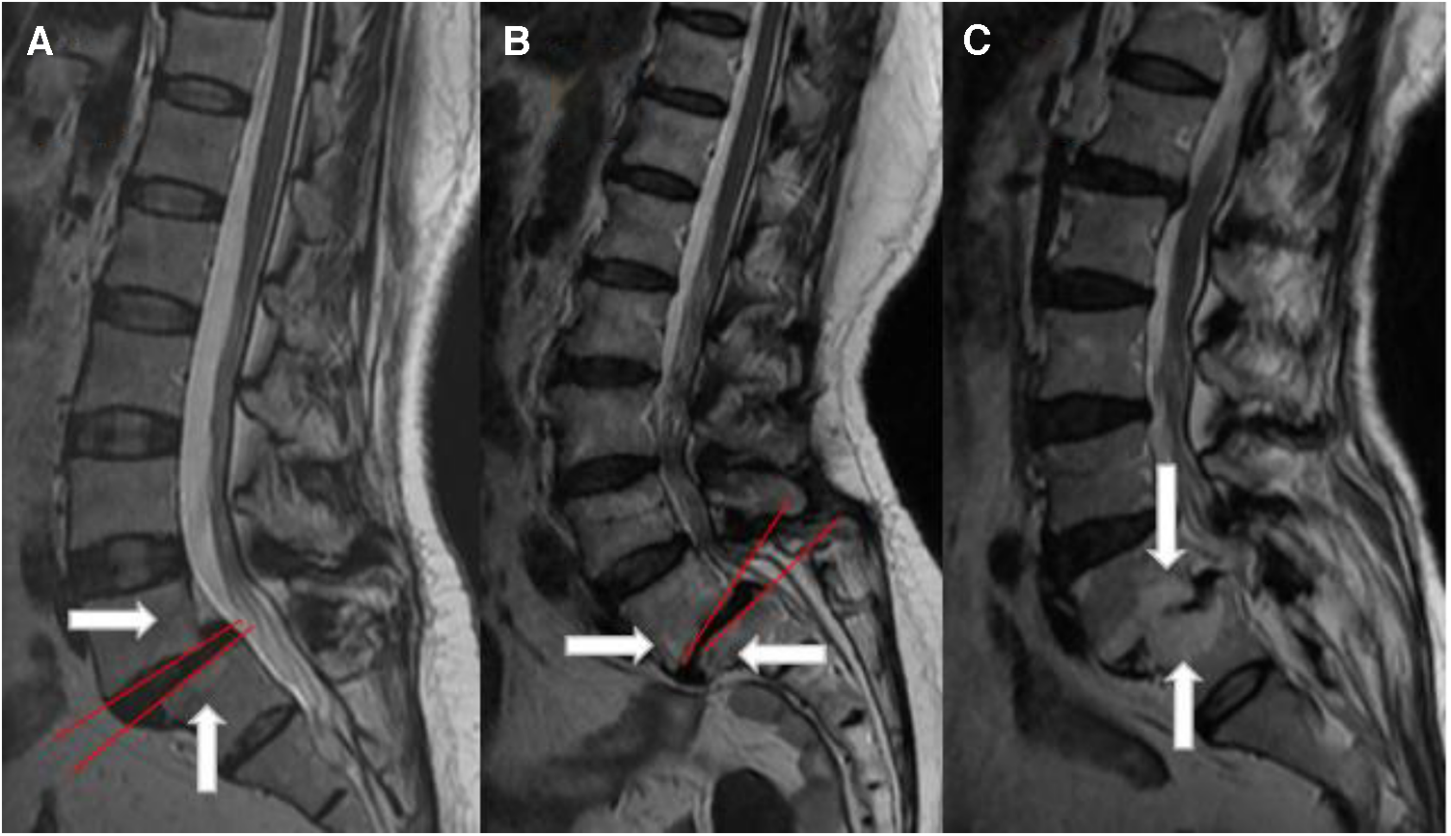
Stress concentration diagram. (**A**) The MDA angle is in the rear, plus its own gravity, and Modic changes often appear in the posterior region of the vertebral body. (**B**) MDA angulation is in the front, and Modic changes often appear in the anterior region of the vertebral body. (**C**) There is no obvious angulation in the spondylolisthesis area, but the intervertebral disc has obvious degeneration, and the intervertebral disc height is significantly reduced. Modic changes are prone to occur in the area opposite the center of the two vertebral bodies, usually type III Modic changes.

The majority of the Modic changes occurred simultaneously in the upper and lower endplate areas, which is consistent with the description of Chen et al. (21). In clinical practice, we found a meaningful phenomenon. After the occurrence of lumbar spondylolisthesis, the displacement of the upper vertebral body relative to the lower vertebral body occurs, and the presence of lumbar lordosis leads to local stress angulation. The angulation is in the rear, and Modic changes are more likely to occur in the rear of the vertebral body. The local activity of partial isthmic spondylolisthesis is extremely large, resulting in stress angulation in the front. In contrast to the lumbar lordosis angle, Modic changes are prone to occur in the front of the vertebral body. If there is no stress angle in lumbar spondylolisthesis, the local intervertebral disc degeneration is serious, and the intervertebral disc height is significantly reduced. Intervertebral disc degeneration is often VI, VII, and VIII grades, and Modic changes often appear in the central corresponding area of the spondylolisthesis vertebral body, which often shows type III Modic changes. According to the above changes and effects, we call them stress concentration effects, as shown in Figure 3.

Logistic regression analysis was used to analyze the risk factors of Modic changes. IDH, MDA, type of spondylolisthesis, degree of spondylolisthesis, and degree of intervertebral disc degeneration were significantly correlated, *P* < 0.05. Isthmic spondylolisthesis is more prone to Modic changes than degenerative spondylolisthesis. Heavier spondylolisthesis, severe intervertebral disc degeneration, lower intervertebral disc height, and the occurrence of local stress angulation are all related to the occurrence of Modic changes.

The limitations of this study are as follows: (1) Cases were only selected from the Fourth People's Hospital of Hengshui City, and other hospitals and medical institutions were not statistically analyzed; it would be robust to conduct statistical analysis of community cases. (2) The sample size is still insufficient. Future studies should consider multicenter involvement and a large sample size to analyze the risk factors of Modic changes in lumbar spondylolisthesis. (3) The stress concentration effect shown in this study was not biomechanically tested. Last but not least, this study is a retrospective case–control study, and higher-level evidence is required in the future.

## Conclusions

Due to the poor local stability of lumbar spondylolisthesis, especially the isthmic type, Modic changes occur. The occurrence of Modic changes is related to the degree of spondylolisthesis, the degree of intervertebral disc degeneration, the decrease of intervertebral disc height, and the angle of local stress.

## Data Availability

The original contributions presented in the study are included in the article/Supplementary Material, further inquiries can be directed to the corresponding author.
